# High volume-rate echocardiography for simultaneous imaging of electromechanical activation and cardiac strain of the whole heart in a single heartbeat in humans

**DOI:** 10.1371/journal.pone.0313410

**Published:** 2024-12-27

**Authors:** Julien Grondin, Hannah J. Schleifer, Rachel Weber, Changhee Lee, Melina Tourni, Elisa E. Konofagou

**Affiliations:** 1 Department of Radiology, Columbia University, New York, NY, United States of America; 2 Department of Biomedical Engineering, Columbia University, New York, NY, United States of America; 3 Department of Neurosurgery, Columbia University, New York, NY, United States of America; Scuola Superiore Sant’Anna, ITALY

## Abstract

**Background:**

Imaging both electrical and mechanical cardiac function can better characterize cardiac disease and improve patient care. Currently, there is no noninvasive technique that can simultaneously image both electrical and mechanical function of the whole heart at the point of care. Here, our aim is to demonstrate that high volume-rate echocardiography can simultaneously map cardiac electromechanical activation and end-systolic cardiac strain of the whole heart in a single heartbeat.

**Method:**

A 32x32 ultrasound matrix array connected to four synchronized ultrasound scanners were used for transthoracic high volume-rate imaging (840 volumes/s) in sixteen young volunteers (28.1±4.2 y.o.). An electromechanical activation map of the whole heart and volumetric end-systolic atrial and ventricular strain images were obtained.

**Results:**

The whole heart activation sequence was found to be consistent across volunteers and in agreement with previously reported normal electrical activation sequences. The mean electromechanical activation time was 72.6±15.2 ms in the atria, 132.4±19.7 ms in the ventricles and 154.5±19.6 ms in the whole heart. Volumetric right and left atrial as well as right and left ventricular strains were also consistent across all volunteers, with a mean end-systolic global longitudinal strain of 26.8±6.5% in the atria and -16.6±3.4% in the ventricles.

**Conclusions:**

This initial feasibility study demonstrates that noninvasive high-volume rate imaging of the heart in a single heartbeat is feasible and can provide electromechanical activation and systolic strains simultaneously in all four cardiac chambers. This technique can be further developed and used at the point of care to assist for screening, diagnosis, therapy guidance and follow-up of heart disease patients.

## Introduction

Electrical and mechanical functions of the heart are interdependent through the excitation-contraction mechanism and the mechano-electric feedback [1]. Therefore, electrical dysfunction can cause mechanical dysfunction and conversely. Coronary artery disease causes blood flow reduction in the coronary arteries, which can lead to myocardial infarction. In addition, an infarct can create life-threatening disturbances of the heart rhythm, such as ventricular tachycardia and ventricular fibrillation. Moreover, left atrial (LA) strain has been shown to be a good predictor of atrial fibrillation [2]. Assessing both electrical and mechanical functions of the heart provides more holistic functional information, which can lead to better patient care. Electrical function is routinely assessed by ECG, but is often performed invasively by electro-anatomical mapping for complex arrhythmia. Here we report simultaneous electromechanical activation mapping and cardiac strain imaging of the whole heart with high-volume-rate single-beat echocardiography. Our group has developed electromechanical wave imaging (EWI), a high frame-rate (500–2000 frames/s) ultrasound-based technique that can map the propagation of the onset of local myocardial shortening which follows local electrical activation [3, 4]. EWI has been validated against electrical mapping in vivo [4, 5] and has been shown to outperform 12-lead ECG in localizing atrial and ventricular arrhythmia [6], localize accessory pathways [7] and differentiate non-responders from super-responders to cardiac resynchronization therapy (CRT) at follow-up [8]. Recently, other groups have developed methods similar to EWI [9–12]. Originally, EWI was developed using 2D echocardiography, however, the 3D propagation of electromechanical waves throughout the complex heart geometry would be better characterized with 3D single-heartbeat imaging. Recently, 3D EWI was developed and validated in silico and in vivo, and feasibility in CRT patients was demonstrated during right ventricular (RV) and Bi-ventricular pacing in good agreement with pacing electrode location [13]. However, single diverging wave imaging was used to reach high volume rate (500–600 volumes/s) and therefore, the image quality was suboptimal compared to conventional focused wave imaging. Diverging wave compounding has been shown to maintain high imaging rate, while significantly improving image quality compared to single diverging wave imaging [14, 15].

On the other hand, cardiac mechanical function in different cardiac chambers can be assessed with cardiac strain imaging using metrics such as global longitudinal strain (GLS) using speckle tracking echocardiography (STE). In the left ventricle (LV), GLS has been reported to improve detection of coronary artery disease [16–18]. RV GLS in patients with heart failure with preserved ejection fraction was found to be lower than in control subjects and to have a strong prognostic value [19]. Peak atrial longitudinal strain was found to be a good predictor of atrial fibrosis in patients undergoing heart transplantation [20]. STE in a specific cardiac chamber can be performed with dedicated echocardiographic views with the chamber in the middle of the field of view. 3D STE can reach a temporal resolution of 20–50 volumes/s with ECG-gated acquisitions by stitching together multiple sub-volumes acquired during multiple (2–7) heartbeats. However, this is inadequate for patients with heart rhythm disorders and patients who cannot hold their breath during the entire acquisition. In addition, current 3D echocardiography temporal resolution may be insufficient for patients with elevated heart rate such as during stress echocardiography or pediatric patients.

Our proposed method differs from STE in that motion tracking is performed on beamformed radiofrequency (RF) ultrasound signals, which leverages phase information (as opposed to B-mode) for optimal tacking resolution and accuracy, together with custom imaging sequences reaching high volume rate imaging (840 volumes/s).

The objective of this study is to show the feasibility of simultaneously imaging electromechanical activation and cardiac strain of the whole heart in humans in vivo in a single heartbeat.

## Methods

### Study design

In this study, 20 healthy young volunteers were recruited for high volume rate echocardiography and scanned between September 1, 2021 and November 30, 2021. Four volunteers (20%) were excluded from the study due to a suboptimal echocardiographic window, and therefore, only 16 subjects (28.1±4.2 y.o., 6 females, 9 males) were analyzed. The study protocol was approved by the institutional review board (IRB) at Columbia University and informed consent was obtained from each participant.

### High-volume rate ultrasound acquisition

**Fig 1:**
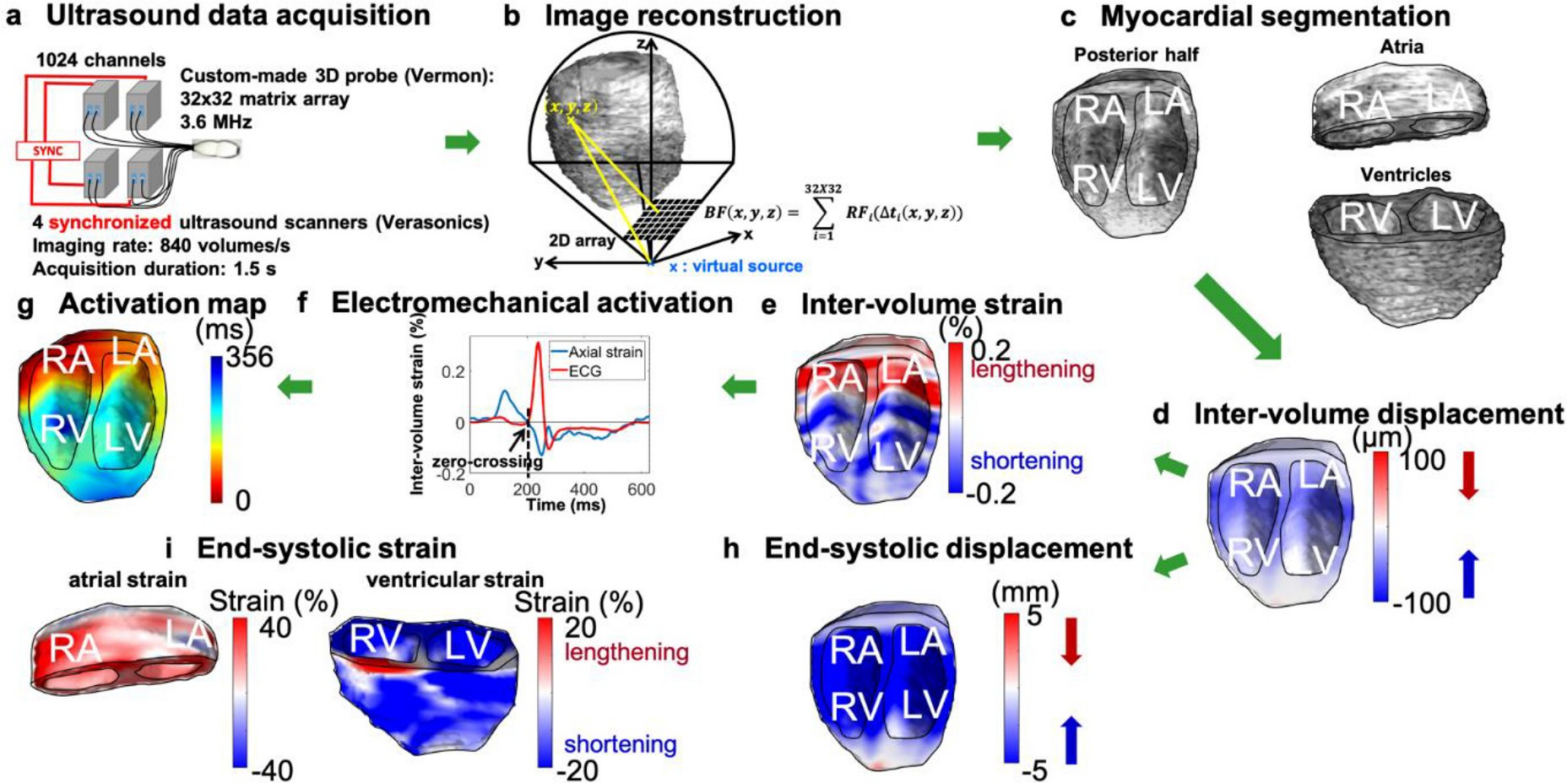
Flowchart of electromechanical wave imaging. Ultrasound data acquisition is performed with a 32x32-element array connected to four synchronized Verasonics ultrasound scanners (a). High volume-rate imaging is performed with diverging wave transmission from 5 virtual sources and 3D image reconstruction is performed with delay-and-sum beamforming (b). The myocardium is manually segmented and the atrio-ventricular junction is used to separate the atria from the ventricles (c). Inter-volume axial displacements are estimated using 1-D normalized cross-correlation of the beamformed radiofrequency ultrasound signals (d). Positive displacements (red) indicate motion towards the transducer (at the apex) and negative displacements (blue) indicate motion away from the transducer. Inter-volume axial strains are estimated using a least-squares estimator (e). Positive strains (red) are associated with longitudinal lengthening and negative strains (blue) are associated with longitudinal shortening. Local onset of contraction is determined by the first positive to negative zero-crossing of the temporal axial strain curve after the onset of the P wave (f). The electromechanical activation map is obtained by determining the onset of contraction in each region of the myocardium (g). Inter-volume displacements are accumulated throughout systole (h). End-systolic strains are computed in the atria and the ventricles (i). LA: left atrium, RA: right atrium, RV: right ventricle, LV: left ventricle.

The ultrasound system consists of four ultrasound scanners (Vantage 256, Verasonics, Kirkland, WA), with 256 channels each and synchronized using a multi-system synchronization module (Verasonics, Kirkland, WA), providing a total of 1,024 channels, which can be used in transmit or receive (Fig 1). A custom-designed 2D array of 32x32 transducer elements (Vermon SA, France), with an inter-element spacing of 0.3 mm and a center frequency of 3.6 MHz was connected to the ultrasound system, therefore, allowing for a 1:1 pin-to-element mapping.

**Fig 2:**
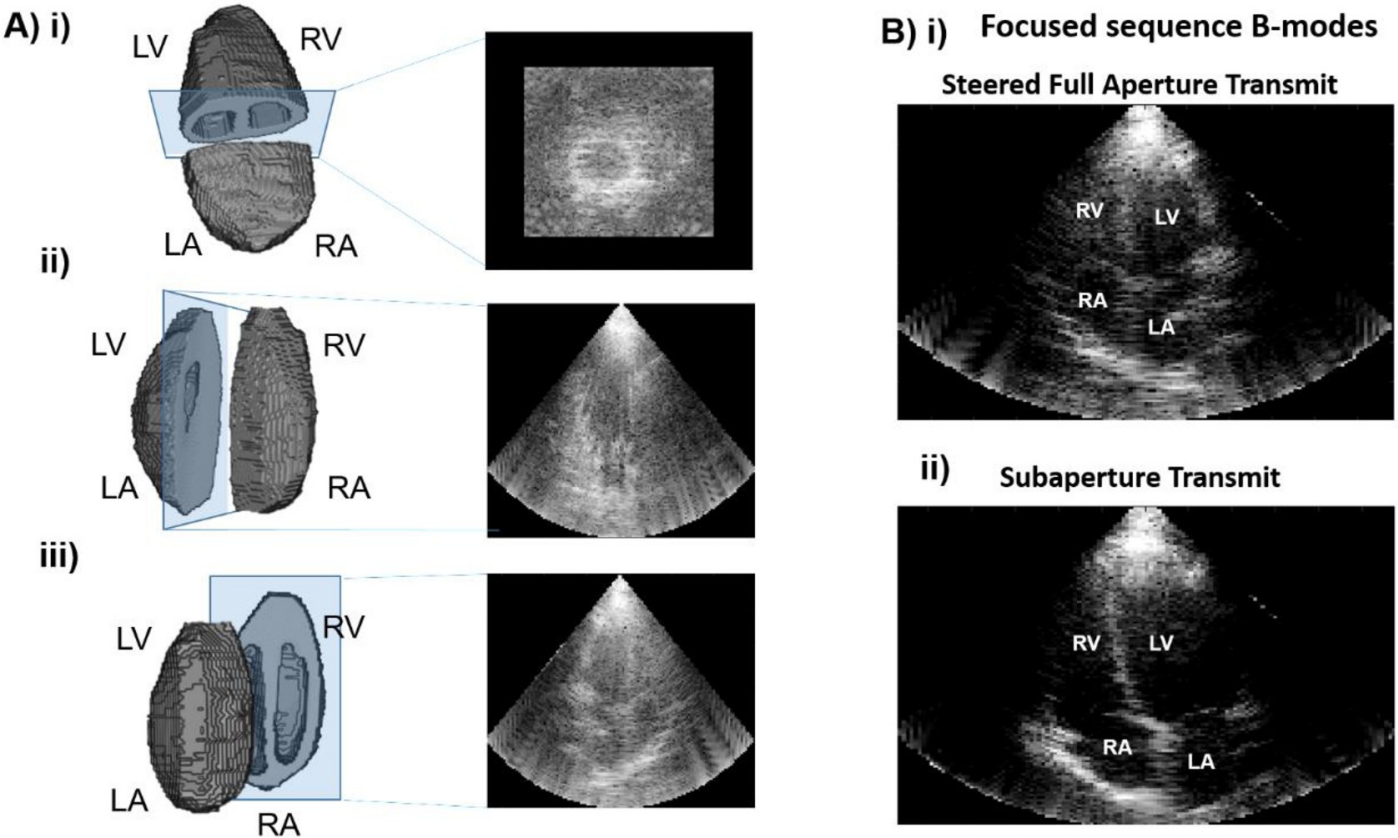
Volumetric imaging B-modes of volunteer 14 (V14). A) i-iii) The 3D B-mode shell and the B-modes reconstructed along the three imaging planes. B) i-ii) The focused B-modes used for quality assessment and segmentation from the steered and the subaperture transmits, respectively.

The ultrasound probe was positioned to acquire apical views of the heart. High volume-rate imaging was achieved by using 3D diverging wave compounding. Ultrasound diverging wave imaging consists of transmitting an ultrasound wave in which the wavefront widens (or diverges) as the wave propagates from the transducer to the tissue. Five different virtual sources were used for diverging wave transmission. Two transmit configurations were used: unsteered diverging wave with sub-aperture transmit (first configuration) and steered diverging wave with full aperture transmit (second configuration). In the first configuration, a 16x16 element sub-aperture located in each quadrant of the 32x32 array as well as one sub-aperture in the center of the array were used. In the second configuration, the transmit steering angles in azimuthal and elevational directions were [+16°, +16°], [+16°, -16°], [-16°, +16°], [-16°, -16°] and [0°, 0°], respectively. While the first configuration allows for a wider virtual aperture and therefore a better spatial resolution, the second configuration allows for a higher transmitted energy and therefore a higher signal-to-noise ratio (SNR). Two acquisitions were performed for each configuration, and only the acquisition with the best image quality out of the four acquired ones was used for post-processing (Fig 2). The pulse repetition frequency was 4200 Hz, yielding an imaging rate of 840 volumes/s after compounding. Delay-and-sum beamforming was used to reconstruct the image in a pyramidal scan format, with an axial sampling of 32.1 μm, a sector angle of 90° and a density of one line per degree in both lateral and elevational directions and was implemented on a GPU (Quadro RTX 8000, NVIDIA, Santa Clara, CA). The beamformed data (BF) at a given location *(x*, *y*, *z)* of the reconstructed volume is given by:

$$BF(x,y,z)=\sum_{j=1}^{5}\sum_{i=1}^{1024}{RF}_{i,j}(\Delta t_{i,j}(x,y,z)){RF}_{i,j}(\Delta t_{i,j}(x,y,z))$$ (1)

where *RF*_*i*,*j*_ is the RF signal received on transducer element *i* from transmit *j* and *Δt*_*i*,*j*_*(x*,*y*,*z)* is sum of the forward delay from the transducer during transmit *j* to the point *(x*,*y*,*z)* and the backward delay from the point *(x*,*y*,*z)* to transducer element *i*. A conventional line-by-line ultrasound acquisition in both azimuthal and elevational directions with a focal depth at 8 cm was performed immediately after the high volume-rate acquisition in order to obtain B-mode images with better quality to assist for volumetric segmentation.

### Myocardial segmentation

The conventional B-mode volume was scan-converted and the myocardium in each cross-section perpendicular to the apex-to-base direction (approximately parallel to the short-axis view) was manually segmented from the apex to the atria. The segmentation was then manually adjusted using the high-volume rate B-mode images and inter-volume strain images because high-volume-rate and conventional B-mode were not obtained at the same time.

### 3D Electromechanical wave imaging of the heart

Normalized cross-correlation [21] (4.9 mm window, 90% overlap) of beamformed RF signals in the axial direction was used to estimate inter-volumes displacements. The axial inter-volume strains were obtained using a least-squares estimator implemented with a Savitzky-Golay filter [22].

By positioning the ultrasound transducer to acquire apical views, the direction of propagation of the ultrasound beam (or axial direction) was approximately aligned with the longitudinal direction of the heart. Therefore, myocardial contraction, which is associated with longitudinal shortening, corresponded to negative axial strain. The onset of longitudinal shortening, resulting from electrical activation, was determined by the time of the first positive-to-negative zero-crossing of the axial strain curves after a reference time point and was defined as the electromechanical activation time. The reference time point was selected as the onset of the P-wave for atrial activation and the onset of the QRS complex for ventricular activation on the simultaneously recorded ECG. The atrio-ventricular (AV) junction was manually selected from the ultrasound B-mode.

Two hundred voxels were randomly selected from the myocardium, and for each voxel, the inter-volume axial strain curve was plotted as well as the ECG. The first positive-to-negative zero-crossing was then manually selected to obtain the electromechanical activation time. In the atria, zero-crossings were considered from the onset of the P-wave to the onset of the Q-wave, while in the ventricle, a time window of approximately 200–300 ms starting from the Q-wave was defined to search for zero-crossings. In some instances, no zero-crossing was found within the time window, but the inter-volume axial strain curve was slightly below zero and then rapidly and strongly decreased and, in this case, the time point before the rapid decrease was selected to define the electromechanical activation time. The electromechanical activation times were then obtained in the entire heart by 3D linear interpolation.

### 3D ventricular and atrial strain imaging

Axial displacement M-mode from the center of the ultrasound volume was used to select the systolic phase, corresponding to an inward motion of the heart. The inter-volume axial displacements were then accumulated over systole. A least-squares estimator was then applied to the accumulated displacements to obtain the axial (longitudinal) strains. The AV junction was used to separately compute atrial and ventricular strains. The atrial (AGLS) and ventricular global longitudinal strain (VGLS) were computed as an average of end-systolic longitudinal strain in the atria (left and right) and the ventricles (left and right), respectively.

### Statistical analysis

The mean and standard deviation, across the 16 volunteers, of the atrial, ventricular and whole heart electromechanical activation times as well as the AGLS and VGLS were computed. All data processing and statistical analysis were performed with MATLAB R2020b (The MathWorks, Natick, MA).

## Results

A custom high-volume-rate transthoracic ultrasound system was developed to image the electromechanical activation of the full heart as well as the full atrial and full ventricular end-systolic strain in a single heartbeat. Diverging wave compounding ultrasound emission was used to achieve high volume rate imaging (840 volumes/s). Inter-volume axial (longitudinal) displacements and strains were estimated. The electromechanical activation map was obtained by determining the local onset of contraction from the inter-volume axial strain curves. Inter-volume displacements were accumulated during systole and systolic strains were obtained with a gradient operator. Average end-systolic strains were computed separately in the atrial and ventricular volumes. The anterior and posterior halves of the heart in the anterior view of the electromechanical activation maps were shown. Anterior and posterior views of the atrial and ventricular volumetric end-systolic strains were shown.

### Electromechanical activation of the full heart

**Fig 3:**
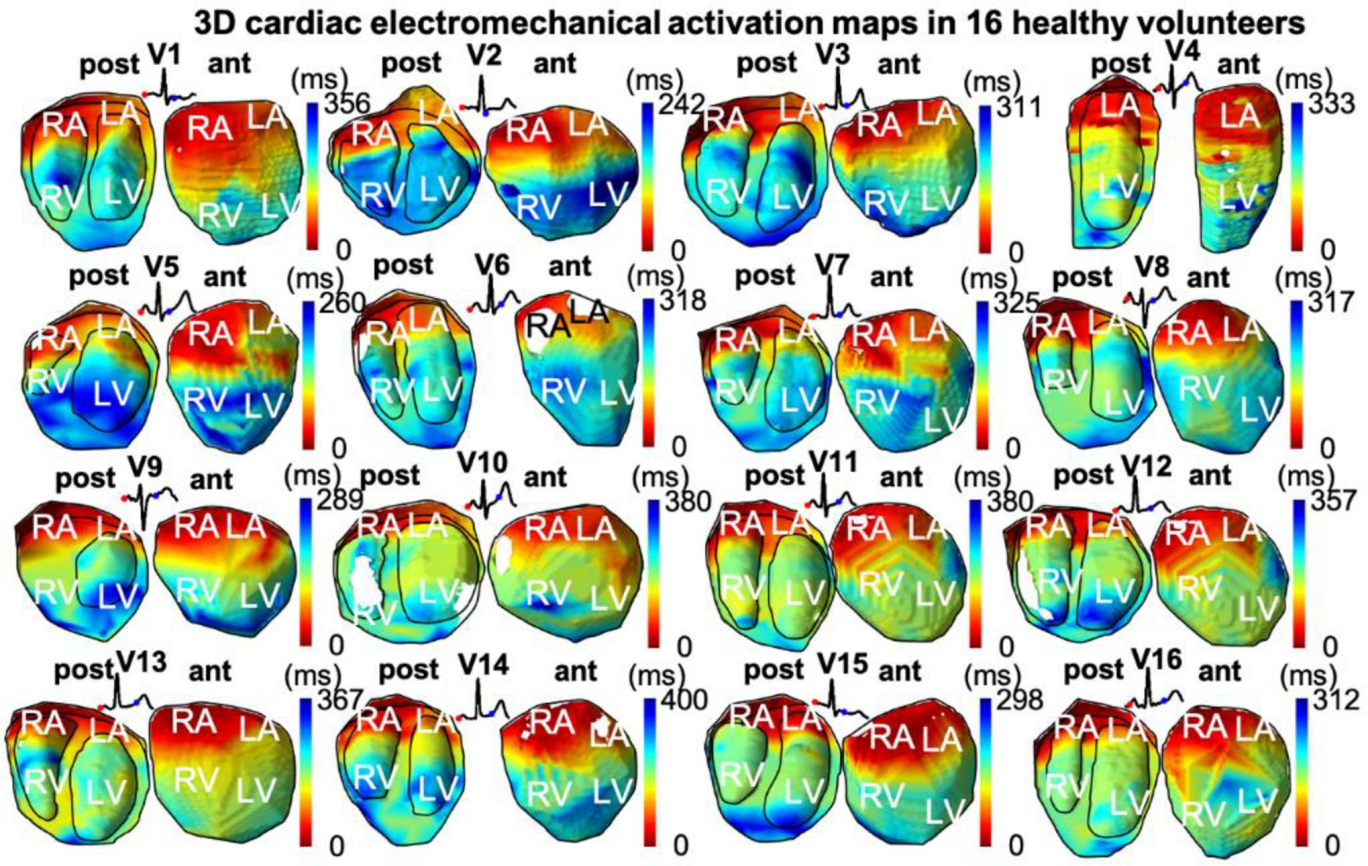
3D electromechanical activation maps in 16 healthy volunteers (V1-V16). Red tones indicate early activation and blue tones indicate late activation. A similar activation pattern is observed in all cases: early activation of the right atrium (RA), followed by the left atrium (LA) followed by left (LV) and right ventricles (RV). Posterior (post) and anterior (ant) halves of the heart are shown. The ECG for each volunteer is shown with earliest and latest EWI activation time points indicated with a red and blue dot, respectively.

Electromechanical activation maps, or isochrones, for all 16 volunteers (V1-V16) during normal sinus rhythm are shown in Fig 3. The earliest activation (in red) was observed in the right atrium (RA), followed by the LA, during the P-wave, and then followed by both ventricles, during and slightly after the QRS complex. Early activation in the ventricles was observed in the middle and lower septal area. Late activation in the ventricles was observed in the posterior-basal region. However, there is an observable variability in the activation pattern across volunteers. The ECG for each volunteer was also shown, with the earliest and latest activation indicated by a red and blue dot, respectively. For volunteer V4, only the left ventricle and atria were fully captured in the field of view. In some volunteers, such as V10 and V12, no electromechanical activation could be detected in some areas, and therefore, these areas were not color-coded. The mean electromechanical activation time across the atria only, the ventricles only, and the whole heart were 154.5±19.6 ms, 132.4±19.7 and 72.6±15.2 ms, respectively (Table 1).

**Table 1:** Mean electromechanical activation times and mean global longitudinal strain in the whole heart averaged across 16 healthy volunteers using single heartbeat high-volume rate ultrasound.

Mean electromechanical activation times			Mean global longitudinal strain	
Atria	Ventricles	Whole heart	Atria	Ventricles
72.6±15.2 ms	132.4±19.7 ms	154.5±19.6 ms	26.8±6.5%	-16.6±3.4%

### Volumetric ventricular global longitudinal strains

**Fig 4:**
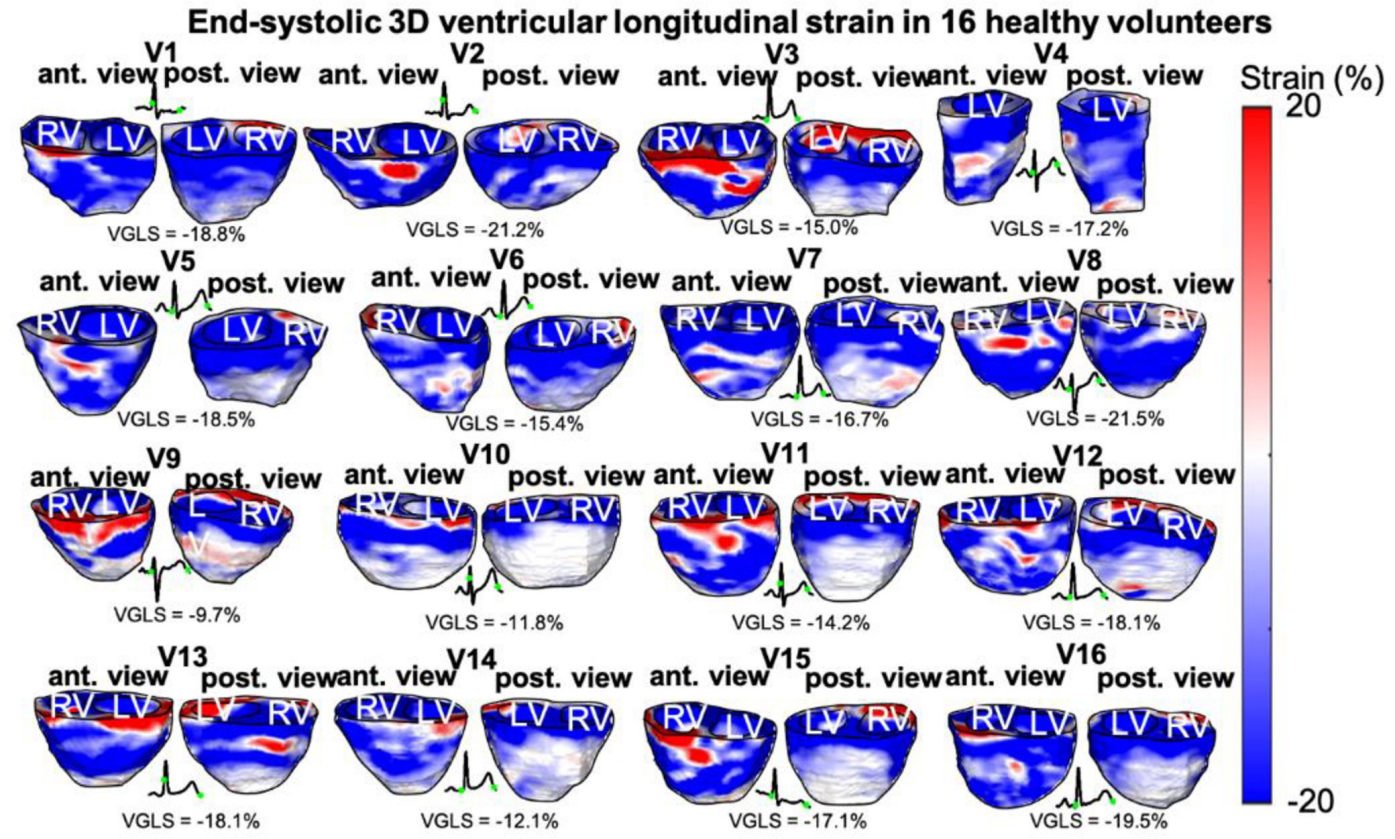
End-systolic 3D ventricular longitudinal strains in 16 healthy volunteers (V1-V16). Ventricular contraction (negative strain) is shown in blue, and lengthening (positive strain) is shown in red. The average global ventricular strain across all volunteers is <VGLS> = -16.6±3.4%. Anterior (ant) and posterior (post) views of the ventricles are shown. The ECG for each volunteer is shown with the beginning and end of systole indicated with a green dot. LV: left ventricle, RV: right ventricle.

Ventricular end-systolic longitudinal strains were imaged for all 16 volunteers (Fig 4). Ventricular contraction (negative strain) is shown in blue, and lengthening (positive strain) is shown in red. All ventricular strain images are shown with the same dynamic range of [-20% 20%]. In general, contraction of both ventricles is observed at end-systole across all volunteers, while some small regions of lengthening are also observed, usually towards the base of the ventricles. In addition, the posterior-apical region of the ventricles tends to have lower strain magnitude compared to the anterior region, such as in volunteers V3, V5, V10-V16. The ECG for each volunteer is shown, and the beginning and end of the systole are indicated by a green dot. For each volunteer, the average end-systolic ventricular (right and left) strain was computed and defined as the ventricular global longitudinal strain (VGLS). The VGLS for each volunteer is shown in Fig 3, and the 95% confidence interval across all volunteers was [-18.3%, -14.8%]. The average VGLS across all volunteers was also computer and was found to be <VGLS> = -16.6±3.4% (Table 1).

### Volumetric atrial global longitudinal strains

**Fig 5:**
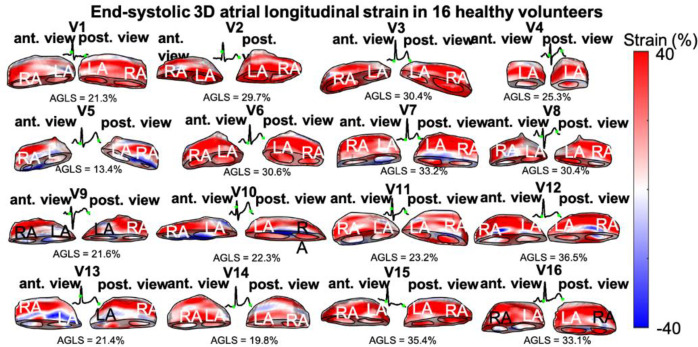
End-systolic 3D atrial longitudinal strains in 16 healthy volunteers (V1-V16). Atrial relaxation (positive strain) is shown in red, and shortening (negative strain) is shown in blue. The average global atrial strain across all volunteers is <AGLS> = 26.8±6.5%. Anterior (ant) and posterior (post) views of the atria are shown. The ECG for each volunteer is shown with the beginning and end of systole indicated with a green dot. LA: left atrium, RA: right atrium.

Atrial end-systolic longitudinal strains were imaged for all 16 volunteers (Fig 5). Atrial relaxation (positive strain) is shown in red, and shortening (negative strain) is shown in blue. All atrial strain images are shown with the same dynamic range of [-40% 40%]. In general, relaxation of both atria is observed at end-systole across all volunteers, while, some small regions of shortening are also observed, usually towards the valves area. Areas of small strain magnitude or negative strain tend to spread along the circumference of the atria. The same ECG as for ventricular strain is shown for each volunteer. For each volunteer, the average end-systolic atrial (right and left) strain was computed and defined as the atrial global longitudinal strain (AGLS). The AGLS is shown for each volunteer in Fig 4, and the 95% confidence interval across all volunteers was [23.3%, 30.2%]. The average AGLS across all volunteers was also computer and was found to be <AGLS> = 26.8±6.5% (Table 1).

## Discussion

Electrical mapping of the heart and its mechanical characterization are performed separately in the clinic, and usually for different clinical purposes. However, the electrical and mechanical functions of the heart are interdependent through the excitation-contraction mechanism and the mechano-electric feedback. Electromechanical wave imaging has been reported to be in good agreement with electrical mapping in pre-clinical and clinical studies as well as in silico. The main aim of this study was to noninvasively image and quantify both electromechanical activation and systolic strain of the whole heart in a single heartbeat. This is of key interest because complex information related to both electromechanical and mechanical cardiac function is captured simultaneously and their relationship can be further elucidated.

In this early feasibility study, we show that the electromechanical activation of the entire heart as well as the volumetric atrial and ventricular longitudinal strain can be simultaneously imaged during a single cardiac cycle. In a previous study, 3D EWI was performed using single diverging wave imaging at a volume rate of 500–600 volumes/s [13]. In this study, we have improved the imaging sequence for better spatial and temporal resolution (840 volumes/s). Improvement in spatial resolution was achieved by compounding five diverging waves, which decreases side lobes and increases SNR. Two different compounding configurations were used (unsteered sub-aperture and steered full aperture). While a one-to-one comparison between the two configurations was not performed due to potential hand motion between acquisitions that can affect image quality, we observed that 14 out of 16 volunteers were best imaged with the steered full aperture configuration. This may be due to higher energy transmitted using the full aperture. The same acquisition data was used to image 3D longitudinal strain in the entire heart. In clinical ultrasound systems, volumetric strain is performed on B-mode images at approximately 20 volumes/s. Therefore, our method shows approximately a 40-fold increase in volume rate.

Similar activation patterns were obtained across all volunteers. The activation sequence was also consistent with our previous studies using 2D and 3D ultrasound [4, 13] as well as with the seminal study of Durrer et al [23]. A perfect agreement is not expected to be obtained due to inter-individual variabilities in the human heart activation sequence [24]. A good agreement was also found between the electromechanical activation map and the ECG, with right-to-left atrial activation during the P-wave and ventricular activation during and slightly after the QRS complex. Electromechanically activated regions after the QRS complex can be due to a longer electromechanical delay.

Ventricular and atrial strains were also relatively consistent across all volunteers, with a mean ventricular and atrial global longitudinal strain of -16.6±3.4% and 26.8±6.5%, respectively. These values are in the same range as what has been reported in the literature using 3D STE. Mean normal LV 3D global longitudinal strains from echocardiography were reported to range from -15.9% to -21% [25–27]. Similarly, RV GLS were reported to be -24.1±3.6% in normal controls [28]. Mean normal 3D echocardiographic LA reservoir (towards end-systole) strain were found to range from 23.7±% to 29.2% [29–31]. Variability in GLS values across different studies can be due to different post-processing algorithms from different vendors. In addition, in our study, 3D GLS was obtained by averaging end-systolic strain from RA and LA on one hand, and RV and LV, on the other hand, which differs from estimating cardiac strain in a single chamber at a time by adjusting the echocardiographic view. A different segmentation method of our dataset would allow for evaluating strain in each cardiac chamber separately. However, we attempted to align the septum wall with the central line of the field of view, to minimize angle-dependency, which can affect strain values. Despite this, areas of strain variability are apparent in the longitudinal strain images, compared to the more consistent electromechanical activation maps. This is expected due to the reported angle-independence of EWI isochrone selection [32]. On the other hand, longitudinal strain magnitude, which is estimated using only axial displacements, is prone to degradation of tracking quality when the direction of myocardial movement is not aligned with the axial dimension of the probe. In the future, employing 2D or 3D cross-correlation searches for displacement estimation is expected to improve angle-independence, as has been shown in the case of 2D myocardial elastography imaging [33].

Reaching high volumetric imaging-rate (840 volumes/s) with diverging wave imaging comes at the cost of decreased contrast and resolution compared to focused wave imaging and can cause suboptimal displacement and strain estimates, especially in lower SNR regions. While coherent compounding of five diverging waves provides higher contrast and resolution than single diverging wave imaging, it does not generate the same image quality as conventionally focused waves.

This initial feasibility study was performed in 20 healthy volunteers, 4 of which were excluded due to suboptimal echocardiographic window precluding adequate segmentation and tracking. A larger study including arrhythmic and ischemic patients will be needed for further validation and to assess the clinical benefit of this combined method.

In this study, manual selection was used to determine electromechanical activation times, which can be subjected to operator bias. A semi-automated method has been developed but may cause higher error rate especially in lower SNR areas. This method can be improved. Another alternative is to use a machine learning based method to automatically determine activation times with a precision that was reported to range from 89% to 97% [34].

One potential application of this combined method would be for treatment planning, treatment monitoring and follow-up of heart failure or irregular arrhythmia patients. Indeed, the relative importance of electrical and mechanical synchrony in CRT patients is still an open question [35, 36] and our technique, which can simultaneously assess electromechanical and mechanical components of the entire heart, can play a major role in improving CRT success. In addition, single-heart-beat 3D activation mapping can largely benefit patients with non-periodic irregular arrhythmias such as atrial fibrillation [37, 38].

Another potential application of our high-volume rate imaging method is for assessing motion abnormalities during stress tests. While under stress, wall motion and deformation abnormalities that are silent at rest may emerge and improve early-stage disease detection [39, 40] however the heart also contracts much more rapidly due to the induced stress. Therefore, temporal resolution higher than currently available in clinical ultrasound scanners is even more critical for patients during stress echocardiography. Currently, a significant number of cardiac disease patients with acceptable echocardiographic windows cannot benefit from 3D strain imaging in the clinic due to insufficient temporal resolution.

Implementation of our technique on a clinical system is feasible. An imaging sequence using diverging wave imaging compounding as opposed to focused beams will need to be used to image the full heart with a high-volume-rate. The implementation of high volume-rate cardiac imaging (820 volumes/s) in a clinical ultrasound system has been reported previously [41], although using four consecutive cardiac cycles with ECG-gated acquisitions.

## Conclusion

This initial feasibility study demonstrates that noninvasive high-volume rate imaging of the full heart in a single heartbeat is feasible and can provide electromechanical activation and systolic strains simultaneously in all four cardiac chambers. A larger study, including cardiac disease patients, will be performed to demonstrate the clinical value of this combined method, such as for heart failure patients. This technique can be used at the point of care and assist for screening, diagnosis and therapy guidance for heart disease patients.

## Supporting information

S1 File IRB approval. This is the approval sent by the Columbia University IRB.
(PDF)

S2 File Approved IRB protocol. This is the approved IRB protocol used to gather data for this work.
(PDF)

## References

[1] Pfeiffer E. R., Tangney J. R., Omens J. H. & McCulloch A. D. Biomechanics of cardiac electromechanical coupling and mechanoelectric feedback. *J Biomech Eng* 136, 021007, doi: 10.1115/1.4026221 (2014). 24337452 PMC4023651

[2] Hauser R. et al. Left atrial strain predicts incident atrial fibrillation in the general population: the Copenhagen City Heart Study. *Eur Heart J Cardiovasc Imaging* 23, 52–60, doi: 10.1093/ehjci/jeab202 (2021). 34632488

[3] Pernot M., Fujikura K., Fung-Kee-Fung S. D. & Konofagou E. E. ECG-gated, mechanical and electromechanical wave imaging of cardiovascular tissues in vivo. *Ultrasound Med Biol* 33, 1075–1085, doi: 10.1016/j.ultrasmedbio.2007.02.003 (2007). 17507146

[4] Provost J., Lee W. N., Fujikura K. & Konofagou E. E. Imaging the electromechanical activity of the heart in vivo. *Proc Natl Acad Sci U S A* 108, 8565–8570, doi: 10.1073/pnas.1011688108 (2011). 21571641 PMC3102378

[5] Grondin J. et al. Validation of electromechanical wave imaging in a canine model during pacing and sinus rhythm. *Heart Rhythm* 13, 2221–2227, doi: 10.1016/j.hrthm.2016.08.010 (2016). 27498277 PMC5079821

[6] Grubb C. S. et al. Noninvasive localization of cardiac arrhythmias using electromechanical wave imaging. *Sci Transl Med* 12, doi: 10.1126/scitranslmed.aax6111 (2020). 32213631 PMC7234276

[7] Melki L. et al. Localization of Accessory Pathways in Pediatric Patients With Wolff-Parkinson-White Syndrome Using 3D-Rendered Electromechanical Wave Imaging. *JACC Clin Electrophysiol* 5, 427–437, doi: 10.1016/j.jacep.2018.12.001 (2019). 31000096 PMC6478397

[8] Melki L. et al. Cardiac Resynchronization Therapy Response Assessment with Electromechanical Activation Mapping within 24 Hours of Device Implantation: A Pilot Study. *J Am Soc Echocardiogr* 34, 757–766.e758, doi: 10.1016/j.echo.2021.02.012 (2021). 33675941 PMC8263475

[9] Andersen M. S. et al. Contractile Fronts In The Interventricular Septum: A Case For High Frame Rate Echocardiographic Imaging. *Ultrasound Med Biol* 46, 2181–2192, doi: 10.1016/j.ultrasmedbio.2020.04.028 (2020). 32561068

[10] Bessière F. et al. High Frame Rate Ultrasound for Electromechanical Wave Imaging to Differentiate Endocardial From Epicardial Myocardial Activation. *Ultrasound Med Biol* 46, 405–414, doi: 10.1016/j.ultrasmedbio.2019.10.017 (2020). 31767455

[11] Christoph J. et al. Electromechanical vortex filaments during cardiac fibrillation. *Nature* 555, 667–672, doi: 10.1038/nature26001 (2018). 29466325

[12] Kvale K. F. et al. Detection of Regional Mechanical Activation of the Left Ventricular Myocardium Using High Frame Rate Ultrasound Imaging. *IEEE Trans Med Imaging* 38, 2665–2675, doi: 10.1109/TMI.2019.2909358 (2019). 30969919

[13] Grondin J., Wang D., Grubb C. S., Trayanova N. & Konofagou E. E. 4D cardiac electromechanical activation imaging. *Comput Biol Med* 113, 103382, doi: 10.1016/j.compbiomed.2019.103382 (2019). 31476587 PMC6817394

[14] Montaldo G., Tanter M., Bercoff J., Benech N. & Fink M. Coherent plane-wave compounding for very high frame rate ultrasonography and transient elastography. *IEEE Trans Ultrason Ferroelectr Freq Control* 56, 489–506, doi: 10.1109/TUFFC.2009.1067 (2009). 19411209

[15] Provost J. et al. 3D ultrafast ultrasound imaging in vivo. *Phys Med Biol* 59, L1–l13, doi: 10.1088/0031-9155/59/19/L1 (2014). 25207828 PMC4820600

[16] Montgomery D. E., Puthumana J. J., Fox J. M. & Ogunyankin K. O. Global longitudinal strain aids the detection of non-obstructive coronary artery disease in the resting echocardiogram. *Eur Heart J Cardiovasc Imaging* 13, 579–587, doi: 10.1093/ejechocard/jer282 (2012). 22166593

[17] Stankovic I. et al. Visual assessment vs. strain imaging for the detection of critical stenosis of the left anterior descending coronary artery in patients without a history of myocardial infarction. *Eur Heart J Cardiovasc Imaging* 16, 402–409, doi: 10.1093/ehjci/jeu206 (2015). 25336543

[18] Uusitalo V. et al. Two-Dimensional Speckle-Tracking during Dobutamine Stress Echocardiography in the Detection of Myocardial Ischemia in Patients with Suspected Coronary Artery Disease. *J Am Soc Echocardiogr* 29, 470–479.e473, doi: 10.1016/j.echo.2015.12.013 (2016). 26852941

[19] Lejeune S. et al. Right Ventricular Global Longitudinal Strain and Outcomes in Heart Failure with Preserved Ejection Fraction. *J Am Soc Echocardiogr* 33, 973–984.e972, doi: 10.1016/j.echo.2020.02.016 (2020). 32387031

[20] Lisi M. et al. Left atrial strain by speckle tracking predicts atrial fibrosis in patients undergoing heart transplantation. *Eur Heart J Cardiovasc Imaging* 23, 829–835, doi: 10.1093/ehjci/jeab106 (2022). 34118154

[21] Luo J. & Konofagou E. A fast normalized cross-correlation calculation method for motion estimation. *IEEE Trans Ultrason Ferroelectr Freq Control* 57, 1347–1357, doi: 10.1109/TUFFC.2010.1554 (2010). 20529710 PMC4123965

[22] Luo J., Bai J., He P. & Ying K. Axial strain calculation using a low-pass digital differentiator in ultrasound elastography. *IEEE Trans Ultrason Ferroelectr Freq Control* 51, 1119–1127, doi: 10.1109/tuffc.2004.1334844 (2004). 15478973

[23] Durrer D. et al. Total excitation of the isolated human heart. *Circulation* 41, 899–912, doi: 10.1161/01.cir.41.6.899 (1970). 5482907

[24] Cardone-Noott L., Bueno-Orovio A., Mincholé A., Zemzemi N. & Rodriguez B. Human ventricular activation sequence and the simulation of the electrocardiographic QRS complex and its variability in healthy and intraventricular block conditions. *Europace* 18, iv4–iv15, doi: 10.1093/europace/euw346 (2016). 28011826 PMC5225966

[25] Bernard A. et al. 3D echocardiographic reference ranges for normal left ventricular volumes and strain: results from the EACVI NORRE study. *Eur Heart J Cardiovasc Imaging* 18, 475–483, doi: 10.1093/ehjci/jew284 (2017). 28329230

[26] Kleijn S. A. et al. Normal reference values of left ventricular strain using three-dimensional speckle tracking echocardiography: results from a multicentre study. *Eur Heart J Cardiovasc Imaging* 16, 410–416, doi: 10.1093/ehjci/jeu213 (2015). 25345661

[27] Muraru D. et al. Left ventricular myocardial strain by three-dimensional speckle-tracking echocardiography in healthy subjects: reference values and analysis of their physiologic and technical determinants. *J Am Soc Echocardiogr* 27, 858–871.e851, doi: 10.1016/j.echo.2014.05.010 (2014). 24975996

[28] Vitarelli A. et al. Three-dimensional echocardiography and 2D-3D speckle-tracking imaging in chronic pulmonary hypertension: diagnostic accuracy in detecting hemodynamic signs of right ventricular (RV) failure. *J Am Heart Assoc* 4, e001584, doi: 10.1161/JAHA.114.001584 (2015). 25792128 PMC4392438

[29] Mochizuki A. et al. Assessment of left atrial deformation and synchrony by three-dimensional speckle-tracking echocardiography: comparative studies in healthy subjects and patients with atrial fibrillation. *J Am Soc Echocardiogr* 26, 165–174, doi: 10.1016/j.echo.2012.10.003 (2013). 23140846

[30] Nabeshima Y., Kitano T. & Takeuchi M. Reliability of left atrial strain reference values: A 3D echocardiographic study. *PLoS One* 16, e0250089, doi: 10.1371/journal.pone.0250089 (2021). 33852637 PMC8046190

[31] Nemes A. et al. Normal reference values of three-dimensional speckle-tracking echocardiography-derived left atrial strain parameters (results from the MAGYAR-Healthy Study). *Int J Cardiovasc Imaging* 35, 991–998, doi: 10.1007/s10554-019-01559-z (2019). 30891666 PMC6534516

[32] Melki L. Costet, A. & Konofagou, E. E. Reproducibility and Angle Independence of Electromechanical Wave Imaging for the Measurement of Electromechanical Activation during Sinus Rhythm in Healthy Humans. *Ultrasound Med Biol*. 43,2256–2268. doi: 10.1016/j.ultrasmedbio.2017.06.019 (2017) 28778420 PMC5562524

[33] Lee W. and Konofagou E. E. Angle-independent and multi-dimensional myocardial elastography—from theory to clinical validation. *Ultrasonics* vol. 48,6–7 doi: 10.1016/j.ultras.2008.07.005 (2008) 18757071 PMC4030389

[34] Melki L., Tourni M. & Konofagou E. E. Electromechanical Wave Imaging With Machine Learning for Automated Isochrone Generation. *IEEE Trans Med Imaging* 40, 2258–2271, doi: 10.1109/TMI.2021.3074808 (2021). 33881993 PMC8410624

[35] Vij A. & Malhotra S. Identifying CRT responders: Moving from electrical to mechanical dyssynchrony. *J Nucl Cardiol*, doi: 10.1007/s12350-022-02914-9 (2022). 35141842

[36] Melki L. et al. A New Electromechanical Wave Imaging Dispersion Metric for the Characterization of Ventricular Activation in Different Cardiac Resynchronization Therapy Pacing Schemes. IEEE Trans. Biomed. Eng. 70(3), 853–859, doi: 10.1109/TBME.2022.3203653 (2023) 36049009 PMC9975111

[37] Costet A. et al. Atrial electromechanical cycle length mapping in paced canine hearts in vivo. IEEE Trans. UFFC 62(7), 1277–1287, doi: 10.1109/TUFFC.2014.006932 (2015) 26168174 PMC4651183

[38] Tourni M. et al. Electromechanical Cycle Length Mapping for atrial arrhythmia detection and cardioversion success assessment. Comp. Biol. Med. 163, 107084, doi: 10.1016/j.compbiomed.2023.107084 (2023) 37302374 PMC10527498

[39] Pellikka P. A. et al. Guidelines for Performance, Interpretation, and Application of Stress Echocardiography in Ischemic Heart Disease: From the American Society of Echocardiography. *J Am Soc Echocardiogr* 33, 1–41.e48, doi: 10.1016/j.echo.2019.07.001 (2020). 31740370

[40] El Harake J. et al. Preliminary Feasibility of Stress Myocardial Elastography for the Detection of Coronary Artery Disease. *Ultrasound in medicine & biology*, 49, 549–559. doi: 10.1016/j.ultrasmedbio.2022.10.007 (2023) 36435662 PMC9789187

[41] Salles S. et al. 3D Myocardial Mechanical Wave Measurements: Toward In Vivo 3D Myocardial Elasticity Mapping. *JACC Cardiovasc Imaging* 14, 1495–1505, doi: 10.1016/j.jcmg.2020.05.037 (2021). 32861651

