# Supplementary material for: High volume-rate echocardiography for simultaneous imaging of electromechanical activation and cardiac strain of the whole heart in a single heartbeat in humans
Source: PLoS One. 2024 Dec 27;19(12):e0313410. doi: 10.1371/journal.pone.0313410 (PMC11676786; doi:10.1371/journal.pone.0313410)
Supplement: S1 File — This is the approval sent by the Columbia University IRB. (PDF) [file pone.0313410.s001.pdf]

Association for the Accreditation of Human Research Protection Programs, Inc.
Full Accreditation

October 24, 2016

Elisa Konofagou
5218101 - ENG BMEN General

Protocol Number: IRB-AAAQ9844
Title: Ultrasonic imaging of patients for cardiovascular studies with Electromechanical Wave Imaging (EWI)
Protocol Version #: 1
Grant #: R01 HL114358
Approval Date: 10/19/2016 Expiration Date: 09/20/2017
Event Identifier: New Protocol (Y01M00)

The above-referenced event was reviewed by Columbia University IRB 3.

Level of review and outcome: Convened IRB review IRB Meeting Date: 09/21/2016

To view a list of documents that were included in this approval (if applicable) and all other currently approved documents for this study, please refer to the Print Menu for this Event in Rascal. It is important to confirm the status of each document, e.g., active, stamped, etc. Only stamped, active documents can be used with research participants.

Consent Requirements:
Informed consent with written documentation will be obtained from the research participant or appropriate representative

HIPAA Authorization:
Authorization will be obtained

Please Note:
1. Spanish consent forms cannot be approved until the revised English form is approved by the Board and translated. Please detach all previously uploaded translations of study documents as these translations must be updated with the requested changes/clarifications provided on 10/17/2016.

Electronically signed by: Collazo, Yaritza

Columbia University IRB
Approved for use until: 09/20/2017

Researcher Responsibilities:

Any proposed changes in the protocol must be immediately submitted to the IRB for review and approval prior to implementation, unless such a change is necessary to avoid immediate harm to the participants.

Any unanticipated problems that involve risks to subjects must be reported to the IRB in accordance with the Unanticipated Problems: Reporting to the IRB of Unanticipated Problems Involving Risks policy. All submissions for modifications and unanticipated problems must be submitted through Rascal.

Renewal applications should be submitted 60 days before the expiration date of this study through Rascal. Failure to obtain renewal of your study prior to the expiration date will require discontinuance of all research activities for this study, including enrollment of new subjects.

You must file a Closure Report in Rascal when your study has been completed.

Columbia University IRB
Approved for
use until: 09/20/2017
