# Supplementary material for: High volume-rate echocardiography for simultaneous imaging of electromechanical activation and cardiac strain of the whole heart in a single heartbeat in humans
Source: PLoS One. 2024 Dec 27;19(12):e0313410. doi: 10.1371/journal.pone.0313410 (PMC11676786; doi:10.1371/journal.pone.0313410)
Supplement: S2 File — This is the approved IRB protocol used to gather data for this work. (PDF) [file pone.0313410.s002.pdf]

# Columbia University Human Subjects Protocol Data Sheet

## General Information

| | | | |
|---|---|---|---|
| **Protocol:** | AAAQ9844(M00Y01) | **Protocol Status:** | Approved |
| **Effective Date:** | 10/19/2016 | **Expiration Date:** | 09/20/2017 |
| **Originating Department Code:** | | ENG BMEN General (5218101) | |
| **Principal Investigator:** | | Konofagou, Elisa (ek2191) | |
| **From what Columbia campus does this research originate:** | | Medical Center | |
| **Title:** | Ultrasonic imaging of patients for cardiovascular studies with Electromechanical Wave Imaging (EWI) | | |
| **Protocol Version #:** | 1 | **Abbreviated Title:** | Cardiovascular imaging with EWI |
| **Was this protocol previously assigned a number by an IRB:** | | | No |

---

**Is the purpose of this submission to obtain a "Not Human Subjects Research" determination?**

No

## Attributes

**Special review type: Check all that apply or check "None of the Above" box.**

[ ]Review for 45 CFR 46.118 Determination (involvement of human subjects is anticipated but is not yet defined)

[ ]Funding review for Administrative IRB approval (such as for Center or Training Grants)

[x]None of the above

---

**IRB of record information: Will a Columbia IRB be the IRB that is responsible for providing review, approval, and oversight for this study?**

Yes

**Select the most appropriate response:**

**Columbia will be the IRB of record for the study procedures conducted by Columbia researchers (Note: this response will apply to most submissions).**

---

**Is this research part of a multicenter study?**

No

---

**Please indicate if any of the following University resources are utilized:**

[ ] Cancer Center Clinical Protocol Data Management Compliance Core (CPDM)

[ ] CTSA-Irving Institute Clinical Research Resource (CRR)

[ ] CTSA- Irving Institute Columbia Community Partnership for Health (CCPH)

[x] None of the above

## Background

**Abbreviated Submission:**

**The IRB has an abbreviated submission process for multicenter studies supported by industry or NIH**

Columbia University IRB
Approved for use until: 09/20/2017

**cooperative groups (e.g., ACTG, HVTN, NCI oncology group studies, etc.), and other studies that have a complete stand-alone protocol. The process requires completion of all Rascal fields that provide information regarding local implementation of the study. However, entering study information into all of the relevant Rascal fields is not required, as the Columbia IRBs will rely on the attached stand-alone (e.g., sponsor's) protocol for review of the overall objectives.**
**If you select the Abbreviated Submission checkbox and a section is not covered by the attached stand-alone protocol, you will need to go back and provide this information in your submission.**

---

**Study Purpose and Rationale:**
**Provide pertinent background description with references that are related to the need to conduct this study. If this is a clinical trial, the background should include both preclinical and clinical data. Be brief and to the point.**

[ ] Abbreviated Submission - This information is included in an attached stand-alone protocol. Proceed to the next question

We hypothesize that Myocardial Elastography (ME) can differentiate normal from pathological myocardium by quantifying its motion, deformation, mechanical and electrical properties.

Detection of cardiac dysfunction through assessment of the mechanical properties of the heart muscle has been a longterm goal in diagnostic cardiology. Ischemia and infarction can successfully be determined through characterization of the regional cardiac function. This is because both ischemia (Haga et al. 1998) and infarction (Bertrand et al. 1978) alter the mechanical properties and contractility of the myocardium. Therefore, alteration of the local myocardial stiffness or of its measured response, such as velocity/displacement, strain rate and strain, could successfully identify pathological regions. In cardiology, the left-ventricular Pressure- Volume curve has been and is still being used extensively for the assessment of the myocardial and chamber compliances in order to detect the acute and chronic changes in the heart muscle as a result of disease (Gaasch et al. 1976). However, apart from the highly invasive nature of these studies, the results from several of them have been either conflicting or representative of a certain type of infarct or ischemia onset (Raya et al. 1988), mainly due to the fact that these techniques are not sensitive to regional changes and, thus, consider the whole myocardium or chamber to undergo uniform motion and deformation; an assumption that does not hold in vivo. Even with experienced physicians and universally accepted methods of diagnosis, such as electrocardiograms, patients still present with ischemia that is undetected by standard clinical examination ([Kukulski et al. 1998], [Dattilo 2008]). These case studies, however, demonstrate that abnormal wall motion undetected by standard imaging techniques and tests were indeed identifiable using strain imaging. Early detection of abnormality is the key to treating cardiovascular disease early and reducing the associated high death toll. The heart, being an inherently dynamically moving organ, alters its mechanical properties as a result of disease. Therefore, current assessment of cardiac wall motion by the routinely used echocardiography in the clinic, relies on qualitative estimation of endocardial wall motion excursion, but is both insensitive and subject to low inter- and intraobserver reproducibility (Solomon et al. 1994).

A study by our group has shown a strong correlation between the Electromechanical Wave (EW), i.e. the early deformation patterns occurring during the QRS complex of the electrocardiogram measured with Myocardial Elastography, and the electrical activation sequence in normal and ischemic animals ([Provost et al., 2009]). An animal study indicated the ability of the method to identify the pacing origin ([Costet et al. 2014]) and its correlation with bipolar electrodes measurement, indicating its potential to not only map the mechanical properties of the myocardium, but also its electrical properties.


Columbia University IRB
Approved for use until: 09/20/2017

Electromechanical Wave Imaging (EWI) is a direct and non-invasive technique capable of mapping the electromechanical activation in all four cardiac chambers in vivo ([Provost et al. 2011]). We refer to ME when we simply focus on the mechanical contraction of the heart, while we use the EWI term when dealing with the electrical conduction aspect and the propagation of the EW wavefront.

Besides, we recently established that EWI is not only capable of properly identifying the origin of activation of focal rhythms, but also of distinguishing between epicardial and endocardial origins in a focal paced canine heart in vivo (Costet et al. 2016 submitted manuscript). These results are very promising, as they could have a major impact clinically for applications in radiofrequency (RF) ablation planning. EWI could therefore constitute a unique tool for the detection and characterization of arrhythmias for screening, diagnosis, treatment planning and monitoring.

Consider a real-time, quantitative assessment of cardiac contractility and electrical activation sequence that can be wheeled into an emergency room, and that can specifically and expeditiously inform a cardiologist or emergency room physician of the status of a patient's heart muscle in different regions of the heart. Potentially, this technology will not only diagnose pathological regions of a patients heart, but also inform caregivers of whether and how well their medical or interventional therapy or even heart transplant is working. This new physiologic data could open up interactive therapy regimes that currently are not considered. The eventual goal of this technology is to become a specific method for estimating the position and severity of contraction and rhythm defects in the myocardium, improving care and outcomes at little more cost or risk than that of a clinical ultrasound.

---

**Study Design:**

**Describe the methodology that will be used in this study, covering such factors as retrospective vs. prospective data collection, interventional vs. non-interventional, randomized vs. non-randomized, observational, experimental, ethnography, etc.**

[ ] Abbreviated Submission - This information is included in an attached stand-alone protocol. Proceed to the next question

The following parameters will be measured in order to assess the ability of cardiac elastography with Electromechanical Wave Imaging to detect and differentiate normal from pathological myocardium: a) specificity (regarding the location of the arrhythmia focus/accessory pathway or of the pacing origin(s)), b) rate of true (sensitivity) and false positives (1- specificity) and c) rate of true and false negatives for each case.

---

**Statistical Procedures:**

**Provide sufficient details so that the adequacy of the statistical procedures can be evaluated including power calculations to justify the number of participants to be enrolled into the study. Definitions of subject terms such as enrolled and accrued as used for Rascal submissions can be found in the Subjects section.**

[ ] Abbreviated Submission - This information is included in an attached stand-alone protocol. Proceed to the next question

The Receiver Operative Characteristic (ROC) curve will be generated given the values of true and false positives and the area under the curve will determine the accuracy of the diagnosis with Electromechanical Wave Imaging.



Columbia University IRB
Approved for use until: 09/20/2017

**Is the purpose of this submission to obtain an exemption determination, in accordance with 45CFR46.101(b):**

No

---

**Is the purpose of this submission to seek expedited review , as per the federal categories referenced in 45CFR46.110?**

No

## Funding

**Is there any external funding or support that is applied for or awarded, or are you the recipient of a gift, for this project?**

Yes

| Award Type | Funding Source Name | Status | Award # or Application Date | Federal/State/Local Government Direct or Subcontract | What is the award covering? | Rascal PT Number |
|---|---|---|---|---|---|---|
| Federal/State/Local Government | National Institute of Health | Awarded/Received | R01 HL114358 | Direct Recipient: No Subcontract Sites | Part of Protocol: Atrial mapping of arrhythmia | PT-AABM9333 |

## Locations

| Location Type | Facility Name | Domestic or International | Geographic Location | Local IRB Ethics Approval | Local Site Approval |
|---|---|---|---|---|---|
| Columbia/CUMC | Herbert Irving Pavillion | | | | |
| Columbia/CUMC | Milstein Hospital | | | | |
| Columbia/CUMC | Neurological Institute | | | | |
| Columbia/CUMC | Presbyterian Hospital | | | | |
| Columbia/CUMC | Vanderbilt Clinic | | | | |

## Personnel

| UNI | Name | Role | Department | Edit/View | Obtaining Informed Consent |
|---|---|---|---|---|---|
| ek2191 | Konofagou, Elisa | Principal Investigator | ENG Biomedical Engineering (521800X) | Edit | N |
| ac3395 | Costet, Alexandre Bernard Emmanuel | Coordinator | ENG Biomedical Engineering (521800X) | Edit | Y |



Columbia University IRB
Approved for use until: 09/20/2017

| UNI | Name | Role | Department | Edit/View | Obtaining Informed Consent |
|---|---|---|---|---|---|
| eab2196 | Bunting, Ethan | Coordinator | ENG Biomedical Engineering (521800X) | Edit | Y |
| es2301 | Silver, Eric | Other Engaged Personnel | PED Cardiology (754050X) | View | Y |
| | **Roles and Experience:** Dr. Silver will provide clinical perspective on the electrophysiology of patients with Wolff Parkinson White Syndrome. He will also assist in obtaining ultrasound images and consenting/assenting his minor patients. | | | | |
| eyw2003 | Wan, Elaine | Other Engaged Personnel | MED Cardiology (751830X) | View | Y |
| | **Roles and Experience:** Dr. Wan will provide clinical perspective on the electrophysiology of patients with heart disease. She will also assist in obtaining ultrasound images. | | | | |
| gar2116 | Rubin, Geoffrey | Other Engaged Personnel | MED Cardiology (751830X) | View | Y |
| | **Roles and Experience:** Dr Rubin, currently in his major clinical year in Cardiology at NYP, is a trained sonographer who will assist in obtaining ultrasound images. | | | | |
| hg2017 | Garan, Hasan | Other Engaged Personnel | MED Cardiology (751830X) | View | N |
| | **Roles and Experience:** Dr. Garan will provide clinical perspective on the electrophysiology of patients with heart disease. Dr. Garan serves as the Dickinson W. Richards, Jr. Professor of Medicine. | | | | |
| jlg2216 | Grondin, Julien | Investigator | ENG Biomedical Engineering (521800X) | Edit | Y |
| | **Roles and Experience:** Julien is a postdoctoral researcher who will assist with the study. | | | | |
| kn2395 | Nakanishi, Koki | Other Engaged Personnel | MED Cardiology (751830X) | View | N |
| | **Roles and Experience:** Koki is a trained sonographer who will assist in obtaining ultrasound images. | | | | |
| ll202 | Liberman, Leonardo | Other Engaged Personnel | PED Cardiology (754050X) | View | Y |
| | **Roles and Experience:** Dr. Liberman will provide clinical perspective on the electrophysiology of patients with Wolff Parkinson White Syndrome. He will also assist in obtaining ultrasound images and consenting/assenting his minor patients. | | | | |
| lm3088 | Melki, Lea | Coordinator | ENG Biomedical Engineering (521800X) | Edit | Y |
| pa2120 | Abreu, Pablo | Coordinator | ENG Biomedical Engineering (521800X) | View | Y |
| | **Roles and Experience:** Administrative Coordinator | | | | |
| vps2109 | Sayseng, Vincent Policina | Coordinator | ENG Biomedical Engineering (521800X) | Edit | Y |

**Training and COI**

**The PI must ensure that each individual that is added as personnel has met the training requirements for this study (http://www.cumc.columbia.edu/dept/irb/education/index.html). For help identifying which research compliance trainings you may be required to take, visit the Research Compliance Training Finder.**

| UNI | Name | COI | HIPAA | HSP (CITI) | Research with Minors (CITI) | FDA-Regulated Research (CITI) | S-I | CRC | Genetic Research Consent |
|---|---|---|---|---|---|---|---|---|---|
| ek2191 | Konofagou, Elisa | 01/12/2016 | 09/13/2004 | 10/27/2015 | 10/16/2009 | 10/27/2015 | | | |



Columbia University IRB
Approved for use until: 09/20/2017

| | | | | | | | | | |
|---|---|---|---|---|---|---|---|---|---|
| ac3395 | Costet, Alexandre Bernard Emmanuel | 11/09/2015 | 07/15/2011 | 07/28/2016 | 07/28/2016 | 07/28/2016 | | 06/17/2015 | |
| eab2196 | Bunting, Ethan | 01/07/2016 | 07/15/2011 | 08/03/2016 | 08/03/2016 | 08/03/2016 | | 06/18/2015 | |
| es2301 | Silver, Eric | 06/24/2016 | 05/30/2008 | 08/17/2015 | 08/17/2015 | 08/17/2015 | | | |
| eyw2003 | Wan, Elaine | 01/07/2016 | 10/19/2006 | 08/04/2016 | 08/04/2016 | 08/27/2014 | | | |
| gar2116 | Rubin, Geoffrey | 04/04/2016 | 06/09/2009 | 09/09/2016 | 06/09/2009 | 09/09/2016 | | 07/12/2016 | |
| hg2017 | Garan, Hasan | 03/04/2016 | 07/02/2004 | 08/30/2014 | 04/26/2006 | 05/26/2011 | | | |
| jlg2216 | Grondin, Julien | 09/02/2016 | 03/21/2012 | 07/30/2016 | 07/30/2016 | 07/30/2016 | | | |
| kn2395 | Nakanishi, Koki | 03/09/2016 | 11/05/2015 | 08/10/2016 | 08/10/2016 | 08/10/2016 | | 11/11/2015 | |
| ll202 | Liberman, Leonardo | 09/02/2016 | 01/03/2006 | 07/31/2015 | 07/31/2015 | 07/31/2015 | | | |
| lm3088 | Melki, Lea | 10/07/2016 | 09/04/2015 | 07/21/2016 | 07/21/2016 | | | 09/04/2015 | |
| pa2120 | Abreu, Pablo | 02/23/2016 | 07/28/2009 | 09/08/2016 | 09/08/2016 | 09/08/2016 | | 06/18/2015 | |
| vps2109 | Sayseng, Vincent Policina | 10/02/2016 | 08/13/2014 | 08/02/2016 | 08/02/2016 | 08/02/2016 | | 06/18/2015 | |

## Departmental Approvers

Electronic Signature: Ethan Bunting (521800X) - Coordinator — Date: 08/11/2016

Electronic Signature: Hasan Garan (751830X) - Other Engaged Personnel — Date: 08/11/2016

Electronic Signature: Lea Melki (521800X) - Coordinator — Date: 08/11/2016

Electronic Signature: Geoffrey Rubin (751830X) - Other Engaged Personnel — Date: 08/26/2016

Electronic Signature: Vincent Policina Sayseng (521800X) - Coordinator — Date: 08/11/2016

Electronic Signature: Eric Silver (754050X) - Other Engaged Personnel — Date: 08/11/2016

Electronic Signature: Pablo Abreu (521800X) - Coordinator — Date: 08/11/2016

Electronic Signature: Elaine Wan (751830X) - Other Engaged Personnel — Date: 08/11/2016

Electronic Signature: Koki Nakanishi (751830X) - Other Engaged Personnel — Date: 08/11/2016

Electronic Signature: Leonardo Liberman (754050X) - Other Engaged Personnel — Date: 08/11/2016

Electronic Signature: Elisa Konofagou (521800X) - Principal Investigator — Date: 10/17/2016

Electronic Signature: Alexandre Bernard Emmanuel Costet (521800X) - Coordinator — Date: 08/11/2016

Electronic Signature: Julien Grondin (521800X) - Investigator — Date: 08/11/2016



Columbia University IRB
Approved for use until: 09/20/2017

## Privacy & Data Security

**Indicate the methods by which data/research records will be maintained or stored (select all that apply):**

[ ]Hardcopy (i.e., paper)

[x]Electronic

**Where will the data be stored?**

Y

[ ]On a System

[x]On an Endpoint

**Identify what type of endpoint will be used (select all that apply):**

[x]Desktop Computer

[ ]Laptop Computer

[ ]Mobile Device

[ ]Other

---

**Does this study involve the receipt or collection of Sensitive Data?**

Yes

**If any Sensitive Data is lost or stolen as part of your research protocol, you must inform both the IRB and the appropriate IT Security Office (CUMC IT Security if at CUMC; CUIT if at any other University campus).**

**What type of Sensitive Data will be obtained or collected? Select all that apply:**

[ ]Personally Identifiable Information (PII), including Social Security Numbers (SSN)

**Will Social Security Numbers (SSNs) be collected for any purpose?**

[x]Protected Health Information (PHI), including a Limited Data Set (LDS)

**If any PHI is lost or stolen, you must inform both the IRB and the Office of HIPAA Compliance.**

**Indicate plans for secure storage of electronic sensitive data: check all that apply**

[ ]Sensitive data will not be stored in electronic format

[ ]Sensitive data will be stored on a multi-user system

[x]Sensitive data will be stored on an encrypted endpoint

**By Selecting an Endpoint Device and approving this protocol for submission to the IRB, the PI is attesting that the device and any removable media that may be used have been or will be registered and/or will be maintained in compliance with the University's Information Security Charter and all related policies. It is important that this information is updated, during the course of the study, as new devices are added.**

---

**Provide a description of how the confidentiality of study data will be ensured, addressing concerns or protections that specifically relate to the data storage elements identified above (e.g. hard copy, electronic, system, and/or endpoint):**

The ultrasound imaging data will be anonymized and stored on a designated secure computer, which will be kept in the Vanderbilt Clinic, 12th Floor, Room 232 behind two locked doors. The hard drives on this computer will be encrypted using Bitlocker drive encryption, and all data files containing patient-sensitive information will be encrypted and password protected.



Columbia University IRB
Approved for use until: 09/20/2017

**Is there or will there be a Certificate of Confidentiality (CoC) for this research?**
No

---

**Provide a description of the protections in place to safeguard participants' privacy while information is being collected:**
Patients will be screened using the iNYP database on a password-protected computer with a secure ethernet connection. Discussion with subjects regarding research participation will take place in a private area (i.e. a patient exam room). All procedures relating to the research study will also be carried out in a private room. Imaging data acquired during research procedures will be anonymized and encrypted. Focus groups will not be used for this study. Subject privacy will be protected as described in attached HIPAA form.

## Procedures

**Is this project a clinical trial?**
No
**Is this project associated with, or an extension of, an existing Rascal protocol?**
Yes
**Existing Rascal protocol #:**
AAAA9333
**Do study procedures involve any of the following?**
**Analysis of existing data and/or prospective record review**
No
**Audio and/or video recording of research subjects**
No
**Biological specimens (collection or use of)**
No
**Cancer-related research**
No
**Drugs or Biologics**
No
**Future use of data and/or specimens**
No
**Genetic research**
No
**Human embryos or human embryonic stem cells**
No
**Imaging procedures or radiation**
Yes
**Medical Devices**
Yes
**Surgical procedures that would not otherwise be conducted or are beyond standard of care**
No
**Will any of the following qualitative research methods be used?**
**Survey/interview/questionnaire**
No


Columbia University IRB
Approved for use until: 09/20/2017

**Systematic observation of public or group behavior**

No

**Program evaluation**

No

**Will any of the following tests or evaluations be used?**

**Cognitive testing**

No

**Educational testing**

No

**Non-invasive physical measurements**

Yes

**Taste testing**

No

**Is there an external protocol that describes ALL procedures in this study?**

No

**Please describe ALL study procedures in detail.**
**NOTE: Be sure to detail all of the procedures above to which a "yes" response was selected. Also detail any additional procedures that may or may not fall into the categories listed above.**

First, RF data will be obtained using a research ultrasound scanner equipped with a 2.5MHz phased array (Vantage 256, Verasonics Inc.) available at the Ultrasound and Elasticity Imaging Laboratory. Then, the data will be 2D envelope-detected (b-scan). Axial displacement and strain estimates will be estimated and imaged using a 1D RF cross correlation algorithm, and activation maps or isochrones can later be generated.

For patients with arrhythmia (ventricular tachycardia, atrial tachycardia, atrial fibrillation, Wolff Parkinson White syndrome...), we will image them at the time of their check-up visit/before their procedure (if planned) and see whether or not we are able to locate the abnormal rhythm origin. In cases such as cardio version or RF ablation, we will image the patients after the procedure in order to compare our findings with the previous results obtained.

For patients with LBBB and atrio-ventricular (A-V) sequential pacemakers and who are not pacemaker dependent, we will first image them with the pacemaker set at the pre-programmer back-up rate and will re-image them while they are paced. For patients with bi-ventricular pacemaker, we will first image the patients using their pre-set A-V and RV-LV delay. We will then re-image them after a standard iterative tissue-Doppler optimization of cardiac re-synchrony. We will further modify A-V and RV-LV delay by incorporating information from the Electromechanical Wave Imaging. Patients will be closely followed subjectively by signs, symptoms and exercise tolerance and objectively by physical exam, hemodynamics, echocardiography, and/or cardio-pulmonary testing. The optimization of cardiac re-synchrony will be within standard set limits of A-V and RV-LV delays; this optimization will not endanger the patients. Imaging will not affect the standard pacing procedure.

## Devices

**On the General Information page you have indicated that the protocol version associated with the use of this medical device is as follows: 1**

**Please note that a Protocol Version # is required for protocols using a medical device, and you will not be allowed to submit this protocol until the Protocol Version # field is complete. Please ensure that the Protocol**



Columbia University IRB
Approved for use until: 09/20/2017

**Version # is completely and accurately reported on the General Information page.**

**Please enter the requested information for each device that is the object of the study or is being used because it is relevant to the aims of the protocol, whether the medical device is not yet FDA-approved [i.e., is investigational] or is an approved device that is being used in an investigational manner (i.e., off-label use is being studied).**

**Note that the questions apply only to devices used in clinical investigations or protocols that involve a Humanitarian Use Device. Emergency use of a device that is not yet FDA-approved is not a clinical investigation, and a submission in Rascal may not be required. Please contact the IRB for assistance if emergency use of a device that is not yet FDA-approved is being considered: (212)305-5883.**

**Device name:**
Vantage
**Device description:**
Research ultrasound system that provides direct access to raw ultrasound under clinical conditions.
**Device Model/Version #:**
Vantage 256
**Phase of Study:**
Pivotal
**Manufacturer Information**

| | |
|---|---|
| **Name:** | Verasonics, Inc. |
| **Address:** | 12016 115th Ave NE, Kirkland, WA 98034, USA |
| **Contact information:** | 425.998.9836; info@verasonics.com |

**Is the device a Humanitarian Use Device (HUD)?**
No
**Is the device FDA-approved and used in accordance with its labeling?**
No
**An Investigational Device Exemption (IDE) may be required.**
**Select Category:**
Not FDA-approved
**Provide plans for storage, control and accounting of the device:**
The device will be stored in the wet lab area of the Ultrasound and Elasticity Imaging Laboratory, P&S 19-418. The device will be used with subjects under the investigator's supervision, and will not be supplied to unauthorized personnel. The serial number of the device will be recorded and connected to the research records of the individual who received the device.
**Is an FDA-issued Investigational Device Exemption (IDE) required?**
No. This is a Nonsignificant Risk device (21 CFR 812.2(b)).
**Will a representative of the Sponsor/Manufacturer be involved with the use of the device at Columbia/ NYPH, e.g., for training purposes?**
No

## Imaging Procedures/Radiation Therapy

**Will a contrast agent (e.g. gadolinium) be used in conjunction with radiation exposure that goes beyond the**


Columbia University IRB
Approved for use until: 09/20/2017

**parameters established for the applicable standard of care (SOC), or will a contrast agent be administered for research purposes only?**
No

**For each type of radiation exposure (e.g., ionizing: CT, X-ray; non-ionizing: MRI), identify the procedure and whether the administration (e.g., radiation dosage, number or type of scans) is clinically indicated and in accordance with the parameters established for the applicable standard of care (SOC), or is "beyond" these parameters (i.e., includes procedures or exposure for research purposes only).**

**Procedure(s) Involving Ionizing Radiation**
**No data to display**

**Procedure(s) Involving Non-Ionizing Radiation**

| Procedure | The exposure to: |
|---|---|
| Ultrasound | Beyond that established for the applicable SOC |

## Recruitment And Consent

### Recruitment:

**Describe how participants will be recruited:**
Patients will be recruited in TWO ways. For both cases, after a verbal description of the study is given by the treating physician, the latter should obtain permission for the study team to contact the patient and document in his medical record that permission was obtained.

Patients recruited will be given the following ICF for the appropriate study:
Method 1 will be given CF-AAAT7723 and Method 2 will be given CF-AAAT6028. In the case of a minor subject for Method 1, CF-AAAT6050 will also be given.

The FIRST method is as follows. In collaboration with the electrophysiology department, arrhythmia patients planned by their healthcare providers for a visit, who are eligible, will be contacted before their procedure. After meeting with the patient and obtaining full consent for our ultrasound study, we will scan the patient prior to, and if possible after their procedure. Our study will not delay or hinder patient medical care, drug delivery, or time to procedure. Patients will still receive appropriate standard of care by their diagnostic and treating physicians regardless of whether they decide to participate in our study. Our ultrasound exam will only take 20 minutes of their time prior to and after their elective procedure and will not intrude or hinder the preparation for the procedure.

The SECOND method is as follows. Patients with LBBB, A-V sequential pacemaker, and bi-ventricular pacemaker will be


Columbia University IRB
Approved for use until: 09/20/2017

recruited for the Cardiac Resynchronization Therapy study. These patients will be recruited from patients admitted to Milstein Hospital, cardiac EP and catheterization laboratory, and cardio-pulmonary testing laboratory in Presbyterian Hospital. Patients will be identified with the assistance of the patient's primary cardiologist at Columbia NYP, heart failure service, cardiology consult service, and arrhythmia service at Milstein Hospital. Patient's involvement in the study is voluntary; patient will need to sign a consent to participate in the study. Our ultrasound exam will only take 40 minutes of their time. Again, the study will not impair, delay, or change the standard of care provided to the patient.

**Select all methods by which participants will be recruited:**

[ ]  Study does not involve recruitment procedures
[x]  Person to Person
[ ]  Radio
[ ]  Newspapers
[ ]  Direct Mail
[ ]  Website
[ ]  Email
[ ]  Television
[ ]  Telephone
[ ]  Flyer/Handout
[ ]  Newsletter/Magazine/Journal
[ ]  ResearchMatch
[ ]  CUMC RecruitMe

---

## Informed Consent Process:

**Informed Consent Process, Waiver or Exemption: Select all that apply**

[x]  Informed consent with written documentation will be obtained from the research participant or appropriate representative.

**Documentation of informed consent is applicable to:**
The study in its entirety

**Identify the portion of the study (e.g., prospective portion, focus groups, substudy 2) or subject population for which documentation of consent will be obtained::**

**Documentation of participation will be obtained from::**

[x]  Adult participants
[x]  Parent providing permission for a child's involvement
[ ]  Legally Authorized Representatives (LARs)

**Describe how participants' written consent will be obtained:**
Written consent from all participants will be obtained on the same day that all research procedures are performed. Each potential subject will be presented with the appropriate consent form. The personnel obtaining consent will provide the subject with a verbal description of the study and consent form.

For minor subjects with Wolff Parkinson White syndrome (in Method 1), on top of written consent from


Columbia University IRB
Approved for use until: 09/20/2017

the parent/guardian, written assent from the minor study participant will be obtained.

If Spanish Speaking Subjects are encountered, the appropriate translation of the the Consent Document (found in the Attachment section of the protocol) will be used to provide an explanation of the study procedures to the subject. The oral explanation will be provided by Pablo Abreu, a bilingual Research Coordinator who is fluent in both English and Spanish. The Consent Form Document will be signed by both the subject and a witness. A copy will be provided to the Non-English Speaking Subject as a Written Summary.

Informed consent is not required for exempt research but is recommended for such research when there will be interaction with research participants for the purpose of the research.

[ ] Informed consent will be obtained but a waiver of written documentation of consent (i.e., agreement to participate in the research without a signature on a consent document) is requested.

[ ] A waiver of some or all elements of informed consent (45 CFR 46.116) is requested.

[ ] Planned Emergency Research with an exception from informed consent as per 21 CFR 50.24.

[ ] Informed consent is not required; this is exempt research.

---

**Subject Language**

Enrollment of non-English speaking subjects is expected.

**Languages anticipated:**

**Spanish**

**As you plan on enrolling non-English speaking subjects, administrative IRB approval of the translated documents (e.g., consent, recruitment materials, questionnaires) in the above selected languages are required. Please see the IRB's policy on the Enrollment of Non-English Speaking Subjects in Research for further details**

**(http://www.cumc.columbia.edu/dept/irb/policies/documents/Nonenglishspeakingsubjects.Revised.FINALDRAFT.111909.website.doc).**

---

**Capacity to Provide Consent:**

**Do you anticipate using surrogate consent or is research being done in a population where capacity to consent may be questionable?**

No

## Research Aims & Abstracts

**Research Question(s)/Hypothesis(es):**

The general objective of this project is to develop a highly reliable and low-cost imaging technique for the early detection of cardiovascular disease based on the resulting altered mechanical



Columbia University IRB
Approved for use until: 09/20/2017

properties of the myocardium walls. The hypothesis behind this study is that the natural contraction of the heart can be used to image displacement and strain of the myocardium using elastographic techniques and is sufficient for the detection of cardiovascular disease at comparable quality to that obtained with the more expensive MRI cardiac tagging techniques and other standard diagnostic imaging techniques, such as coronary angiography. It is further hypothesized that the resulting image quality is comparable to that obtained with other more established imaging techniques, such as 2D transthoracic echocardiography and intracardiac echocardiography. This technique can also be applied non-invasively for the assessment of treatment of disease such as cardiac resynchronization therapy or PVC ablations. It can also be applied minimally invasively with the use of an intracardiac echocardiography catheter.

---

**Scientific Abstract:**

The general objective of this project is to develop a highly reliable and quantitative imaging technique for the early detection of cardiovascular disease based on the resulting altered mechanical and electrical properties of the myocardium and arterial walls. The hypothesis behind this study is that the natural deformation the myocardium undergoes can be used to image displacement and strain of the cardiovascular tissue at comparable quality to that of MRI tagging and other routine cardiovascular diagnostic techniques.

A study from our group (Preliminary validation of angle-independent myocardial elastography using MR tagging in a clinical setting, Lee et al. 2008) has already proven elastography capable of estimating myocardial deformation in good agreement with tMRI estimates in a clinical setting and of differentiating abnormal from normal myocardium in a full left ventricular view.

We hereby propose to explore the clinical potential of Elastography in the detection of cardiovascular disease and arrhythmia and hypothesize that Electromechanical Wave Imaging can be used to differentiate abnormal conduction patterns from normal sinus rhythm, and detect the origin location of abnormal rhythms.

The specific aim is to apply Myocardial Elastography with Electromechanical Wave Imaging (EWI) on subjects with cardiovascular disease in vivo and compare it to clinically standard techniques. For instance, the EWI outcomes on CRT patients regarding the optimal pacing configuration of the pacemaker can be compared with the results given by the metric of left ventricle stroke volume. As for arhythmic patients, earlier origins of activation found with EWI results will be compared to the invasive and time consuming electrophysiology mapping conclusions.

Imaging of the strain within the myocardial ultrasonic signal represents a significant improvement over current methods for assessing myocardial contractility and arrhythmia. The eventual goal of this technology is to become a specific method in standard clinical practice for real-time imaging of the position and severity of contraction and electrophysiological defects in cardiovascular disease, improving care and outcomes at little more cost or risk than that of a clinical ultrasound.

---

**Lay Abstract:**

Early detection of cardiovascular diseases has been an important area of research in the medical imaging field. Assessment of the regional and global function of the heart is very important for accurate diagnosis. Traditionally, elastography is a method for analyzing ultrasonic signals acquired before and after an applied compression to image tissue elasticity. Elastography has proven clinically useful and has been shown to play an additive role to regular ultrasound for breast and prostate cancer detection. We have shown that similar ideas can be applied to ultrasonic



Columbia University IRB
Approved for use until: 09/20/2017

assessment of cardiac tissue, and have demonstrated that elastography for estimation of the local cardiac muscle displacements and strain in vivo can be successfully accomplished. Identification of regions of myocardial dysfunction remains a challenge in echocardiography. Most current methods for detection of regional myocardial dysfunction rely on visual tracking of the endocardial border and observation of myocardial thickening. Nevertheless, these methods remain subjective and imprecise, and it can be difficult to distinguish endocardial motion of the heart from myocardial contraction. In this study, we propose to explore the role of elastography in the detection of cardiovascular disease. We hope to show that, since cardiac elastography and Electromechanical Wave Imaging use the same data as echocardiography, they can offer the precision and accuracy of more expensive and longer tests, such as angiography and electrophysiological mapping, at lower financial and temporal costs.

## Risks, Benefits & Monitoring

**Abbreviated Submission:**
**The IRB has an abbreviated submission process for multicenter studies supported by industry or NIH cooperative groups (e.g., ACTG, HVTN, NCI oncology group studies, etc.), and other studies that have a complete stand-alone protocol. The process requires completion of all Rascal fields that provide information regarding local implementation of the study. However, entering study information into all of the relevant Rascal fields is not required, as the Columbia IRBs will rely on the attached stand-alone (e.g., sponsor's) protocol for review of the overall objectives. .**
**If you select the Abbreviated Submission checkbox and a section is not covered by the attached stand-alone protocol, you will need to go back and provide this information in your submission.**

---

**Potential Risks:**
**Provide information regarding all risks to participants that are directly related to participation in this protocol, including any potential for a breach of confidentiality. Risks associated with any of the items described in the Procedures section of this submission should be outlined here if they are not captured in a stand-alone protocol. Risks of procedures that individuals would be exposed to regardless of whether they choose to participate in this research need not be detailed in this section, unless evaluation of those risks is the focus of this research. When applicable, the likelihood of certain risks should be explained and data on risks that have been encountered in past studies should be provided.**

[ ] Abbreviated Submission - This information is included in an attached stand-alone protocol. Proceed to the next question

The Verasonics scanner is not an FDA approved device, though hard limits have been set to ensure ultrasound emissions well within FDA limits. The following safety protocol has been designed to ensure patient safety:

In order to test the acoustic output of our ultrasound system, a standard experimental setup similar to that described in Harris (see attached) was used. The ultrasound probe (P4-2 ATL phased array) was suspended in a degassed water bath with the transducer face oriented towards a hydrophone (Onda HNP-0200), a highly-sensitive device capable of measuring ultrasound pressures. The hydrophone was positioned in the spatial peak of the pressure field, and pressure measurements were digitized and saved onto a computer for post-processing. Pressure measurements were recorded for all possible output levels. The pressures recorded are related to both the Ispta and MI, which were calculated from these measurements. This experiment was


Columbia University IRB
Approved for use until: 09/20/2017

performed by two operators, who obtained similar results.

Having obtained a calibration between output level (1-50) and acoustic intensity, limits were incorporated into the Verasonics user interface. In detail, the user must choose a beam formation when he/she starts the scanner. Calibration data is hard-coded in the ultrasound scanner, so the user will only be able to apply output levels from 0 to within 90% of the FDA limits (Ispta of 430 mW per cm squared and MI of 1.9). The method for choosing power will be a slider, which scales from 0 to the calibration limit, so the user will be incapable of applying output levels beyond the slider limit. Because two parameters are limiting the output, Ispta and MI, the most restrictive limit will be used as our hardcoded output limit. In addition, a redundant check will be implemented, so that if the power level exceeds the calibration limit, a warning will be displayed, and the scanner will not emit any acoustic radiation. For example, if for a particular beam formation, the output level corresponding to the Ispta limit is 22, while the output level corresponding to the MI limit is 34. Since the user interface limits the output levels to 90% of the FDA limits and the Ispta limit is the most restrictive for this case, the user will be able to choose output levels between 0 and 19.8 (producing and Ispta between 0 and 387 mW/cm squared and an MI between 0 and 1.3). During clinical ultrasound acquisition, a sonographer will operate the ultrasound probe, while a member of our team will operate the user interface on the Verasonics scanner.

The user interface raw code will be stored and modified on a separate computer. Calibrations cannot be directly altered on the scanner, to avoid accidental alterations. We will implement calibration checks on a bi-annually basis using the experimental setup described above. The new calibrations will be compared to the calibrations implemented on the system, and the system calibrations will be updated. The only way to override the calibration limits will be to alter the code of the user interface (stored and maintained on a separate computer), and Vincent Sayseng will be the only member authorized to alter this user interface or the embedded calibration data.

The IND/IDE Assistance Program has reviewed this strategy, and has determined that the device and its proposed use to be of non-significant risk (NSR), and no IND/IDE number is necessary.

---

**Potential Benefits:**

**Provide information regarding any anticipated benefits of participating in this research. There should be a rational description of why such benefits are expected based on current knowledge. If there is unlikely to be direct benefit to participants/subjects, describe benefits to society. Please note that elements of participation such as compensation, access to medical care, receiving study results, etc. are not considered benefits of research participation.**

[ ] Abbreviated Submission - This information is included in an attached stand-alone protocol. Proceed to the next question

This study can yield an early-detection, noninvasive or minimally invasive and low-cost imaging technique using currently available imaging systems.

---

**Alternatives:**

**If this research involves an intervention that presents greater than minimal risk to participants, describe available alternative interventions and provide data to support their efficacy and/or availability. Note, participants always have the option not to participate in research.**

[ ] Abbreviated Submission - This information is included in an attached stand-alone protocol. Proceed to the next question


Columbia University IRB
Approved for use until: 09/20/2017

The only alternative is not to participate in the study.

---

**Data and Safety Monitoring:**

**Describe how data and safety will be monitored locally and, if this is a multi-center study, how data and safety will be monitored across sites as well.**

[ ] Abbreviated Submission - This information is included in an attached stand-alone protocol. Proceed to the next question

To ensure patient safety, the beam forming and user interface raw code will be reviewed on a monthly basis.

Besides, Research Coordinators will perform calibration checks bi-annually and if necessary calibration thresholds will be updated to remain within the Ispta and MI FDA regulation limits.

## Subjects

**Unless otherwise noted, the information entered in this section should reflect the number of subjects enrolled or accrued under the purview of Columbia researchers, whether at Columbia or elsewhere.**

**Target enrollment:**

100

**Number anticipated to be enrolled in the next approval period:**

40

**Does this study involve screening/assessment procedures to determine subject eligibility?**

No

**Is this a multi-center study?**

No

**Does this study have one or more components that apply to a subset of the overall study population (e.g. Phase 1/2, sub-studies)?**

No

**Target Enrollment Demographics:**

**Population Gender**

| Females | Males | Non Specific |
|---|---|---|
| 0% | 0% | 100% |

**Population Age**

| 0-7 | 8-17 | 18-65 | >65 | Non Specific |
|---|---|---|---|---|
| 0% | 15% | 45% | 40% | 0% |

**Population Race**

| American Indian/Alaskan Native | Asian | Native Hawaiian or Other Pacific Islander | Black or African American | White | More than One Race | Non-Specific |
|---|---|---|---|---|---|---|
| 0% | 0% | 0% | 0% | 0% | 0% | 100% |

**Population Ethnicity**

| Hispanic or Latino | Not Hispanic or Latino | Non-Specific |
|---|---|---|



Columbia University IRB
Approved for use until: 09/20/2017

| | | |
|---|---|---|
| 0% | 0% | 100% |

**Vulnerable Populations as per 45 CFR 46:**

**Will children/minors be enrolled**

Yes

**Note that upon "Save", you will see a link to the required "Child Involvement" page in the left side navigation menu. You must complete this page prior to submission.**

**Will pregnant women/fetuses/neonates be targeted for enrollment?**

No

**Will prisoners be targeted for enrollment?**

No

**Other Vulnerable Populations:**

[ ]Individuals lacking capacity to provide consent
[ ]CU/NYPH Employees/Residents/Fellows/Interns/Students
[ ]Economically disadvantaged
[ ]Educationally disadvantaged
[x]Non-English speaking

**Please ensure that your plan to enroll subjects in their primary language is described on the Informed Consent page.**

[ ]Other Vulnerable populations
[ ]None of the Populations listed above will be targeted for Enrollment

**Subject Population Justification:**

Patients with cardiac disease will be recruited for sample size calculations and future inferential studies regardless of age, gender or ethnicity. A two-group power analysis was performed on preliminary data in order to determine the optimal number of subjects. From previous recruitment methods, about 30% of consenting subjects were eligible for absolute inclusion. Taking into account this inclusion rate, our analysis yielded the following estimates for the total number subjects to be recruited: 10 subjects with a pacemaker, 20 subjects with a bi-ventricular pacemaker, 10 patients with atrial flutter, 15 patients with atrial fibrillation, 15 patients with ventricular tachycardia, 15 patients with atrial tachycardia, 15 minors with Wolff Parkinson White (WPW) syndrome.

Children were chosen for our arrhythmia study, since WPW is more common in minors as it hasn't been treated yet.

**Does this study involve compensation or reimbursement to subjects?**

No

## Child Involvement

**RISK/BENEFIT DETERMINATION**

**Please refer to the Columbia University IRB policy on research involving children for further information. (Available on the IRB websites: CUMC IRB or Morningside/LDEO IRB.)**

**'Minimal risk' means that the probability and magnitude of harm or discomfort anticipated in the research are not greater in and of themselves than those ordinarily encountered in daily life or during the performance of routine physical or psychological examinations or tests.**

**Select the option below that best describes your study.**

No more than Minimal Risk (45 CFR 46.404/21 CFR 50.51; i.e., 'Section 404')

**Explain how the risks of the research are minimal. 'Minimal Risk' means that the probability and magnitude of**



Columbia University IRB
Approved for use until: 09/20/2017

**harm or discomfort anticipated in the research are not greater in and of themselves than those ordinarily encountered in daily life or during the performance of routine physical or psychological examinations or tests.**
The minors will only be getting ultrasound scans, which are non-invasive and non-ionizing. These patients are ordinarily scanned by their healthcare providers at the time of their visit.

**WARDS AND FOSTER CHILDREN**

**If 'Section 406' or 'Section 407' research was indicated, the inclusion of wards or foster children requires additional information and, if the research will be conducted in New York City (NYC), approval from the NYC Administration for Children's Services (ACS). Please select the appropriate option below.**
This research has not been categorized as 45 CFR 46.406 ('Section 406') or 45 CFR 46.407 ('Section 407').

**ASSENT OF SUBJECTS**
**Assent of the child is required except in limited circumstances. The first step in determining whether assent is required and/or appropriate is to assess whether the children who will participate in the study will be capable of providing assent. The next step is to determine, for children who are capable of providing assent, whether assent will be obtained or should be waived.**

**Indicate whether the children who will be enrolled in this study will generally be capable of providing assent.**
Some or all are expected to be capable of providing assent.

**Please explain why some or all of the children are expected to be capable of providing assent, and if applicable, why some may not be capable.**
The children will be aged 10 and above, will be in a normal psychological state, and will possess full cognitive abilities.
**For the children who are capable of providing assent, indicate whether you propose to obtain assent or to request a waiver of the requirement to obtain assent.**
Assent will be obtained from children who are capable of providing voluntary and informed agreement to participate.

**Describe the process that will be used (e.g., with or without parents present, whether models, diagrams, or other aids will be used).**
Written assent will be obtained on the same day that all research procedures are performed. The parent(s)/legal guardian(s) will be present and one parent will at the same time have to sign a consent form.
**Describe how assent will be documented (e.g., signed assent form, verbal assent with documentation of process in the research record).**
Written assent will be obtained and the signed assent forms will be kept for documentation.

**PARENT/GUARDIAN PERMISSION**
**Permission of parents/guardians of the children is required except in limited circumstances. Permission from one parent/guardian is acceptable for research categorized as Section 404 or Section 405 unless waiver of informed consent is approved or the IRB determines that permission from both parents is warranted.**

**Select the parental permission option that applies to your study, and provide the rationale for your response if justification is requested. For most studies, one selection is appropriate, however, if more than one option applies, select all that apply.**
[x] The permission of one parent/guardian will be obtained.
[ ] The permission of both parents/guardians will be obtained. - THIS IS REQUIRED IF YOU HAVE CATEGORIZED YOUR RESEARCH AS 45 CFR 46.406 OR 45 CFR 46.407
[ ] No parental permission will be obtained because each of the following waiver criteria for waiving parental permission apply (45 CFR 46.408(c)):
[ ] No parental permission will be obtained because the involvement of children in this research meets the criteria for a

Columbia University IRB
Approved for use until: 09/20/2017

complete waiver of consent (45 CFR 46.116(d)), which is requested in the “Recruitment and Informed Consent” section.

## Attached HIPAA Forms

| Number | Type | Title | Status |
|---|---|---|---|
| AAAN4064 | A | EWI | Approve |
| AAAN4067 | A | EWI Spanish | Approve |
| AAAN4715 | D | Pre-Research HIPAA EWI | Approve |

## Attached Consent Forms

| Number | Copied From | Form Type | Title | Active/InActive | Initiator |
|---|---|---|---|---|---|
| AAAT6028 | | Consent | EWI with CRT device | Active | Lea Melki (lm3088) |
| AAAT6032 | | Consent | EWI General without CRT | Inactive | Lea Melki (lm3088) |
| AAAT6050 | | Assent | EWI general kids | Active | Lea Melki (lm3088) |
| AAAT7723 | AAAT6032 | Consent | EWI General without CRT | Active | Lea Melki (lm3088) |

## Documents

| Archived | Document Identifier | Document Type | File Name | Active | Stamped | Date Attached | CreatedBy |
|---|---|---|---|---|---|---|---|
| No | Spanish assent form | Assent Form | CF-AAAT6050-Spanish.pdf | Y | | 10/02/2016 | Lea Melki (lm3088) |
| Yes | Spanish assent form | Assent Form | CF-AAAT6050-Spanish.pdf | Y | | 08/12/2016 | Lea Melki (lm3088) |
| No | Spanish Certification | Certificate of Translation | Spanish Certification.pdf | Y | | 08/12/2016 | Lea Melki (lm3088) |
| No | Spanish consent form AAT6028 | Consent Form/Addendum | CF-AAAT6028-Spanish.pdf | Y | | 10/02/2016 | Lea Melki (lm3088) |
| Yes | Spanish consent form AAAT6028 | Consent Form/Addendum | CF-AAAT6028-Spanish.pdf | Y | | 08/12/2016 | Lea Melki (lm3088) |
| Yes | Spanish consent form AAAT6032 | Consent Form/Addendum | CF-AAAT6032-Spanish.pdf | Y | | 08/12/2016 | Lea Melki (lm3088) |
| No | Spanish consent form AAT7723 | Consent Form/Addendum | CF-AAAT7723-Spanish.pdf | Y | | 10/02/2016 | Lea Melki (lm3088) |
| No | FDAregulations-Harris | FDA Form/Documentation | FDAregulations.pdf | Y | | 07/26/2016 | Lea Melki (lm3088) |
| No | R01 HL114358-03 Progress Report | Funding/Grant Application/Subcontract | R01_HL114358_03.pdf | Y | | 07/26/2016 | Lea Melki (lm3088) |
| No | R01 HL114358-04 Progress Report | Funding/Grant Application/Subcontract | R01_HL114358_04.pdf | Y | | 07/26/2016 | Lea Melki (lm3088) |
| No | Verasonics Vantage Device Brochure | Investigator Brochure/Package Insert/Device Manual | Verasonics Vantage Family Brochure and Specifications(1).pdf | Y | | 07/26/2016 | Lea Melki (lm3088) |



Columbia University IRB
Approved for use until: 09/20/2017
